# Supplementary material for: NMDAR-dependent long-term depression is associated with increased short term plasticity through autophagy mediated loss of PSD-95
Source: Nat Commun. 2021 May 14;12:2849. doi: 10.1038/s41467-021-23133-9 (PMC8121912; doi:10.1038/s41467-021-23133-9)
Supplement: Supplementary file 1 — Supplementary Information [file 41467_2021_23133_MOESM1_ESM.pdf]

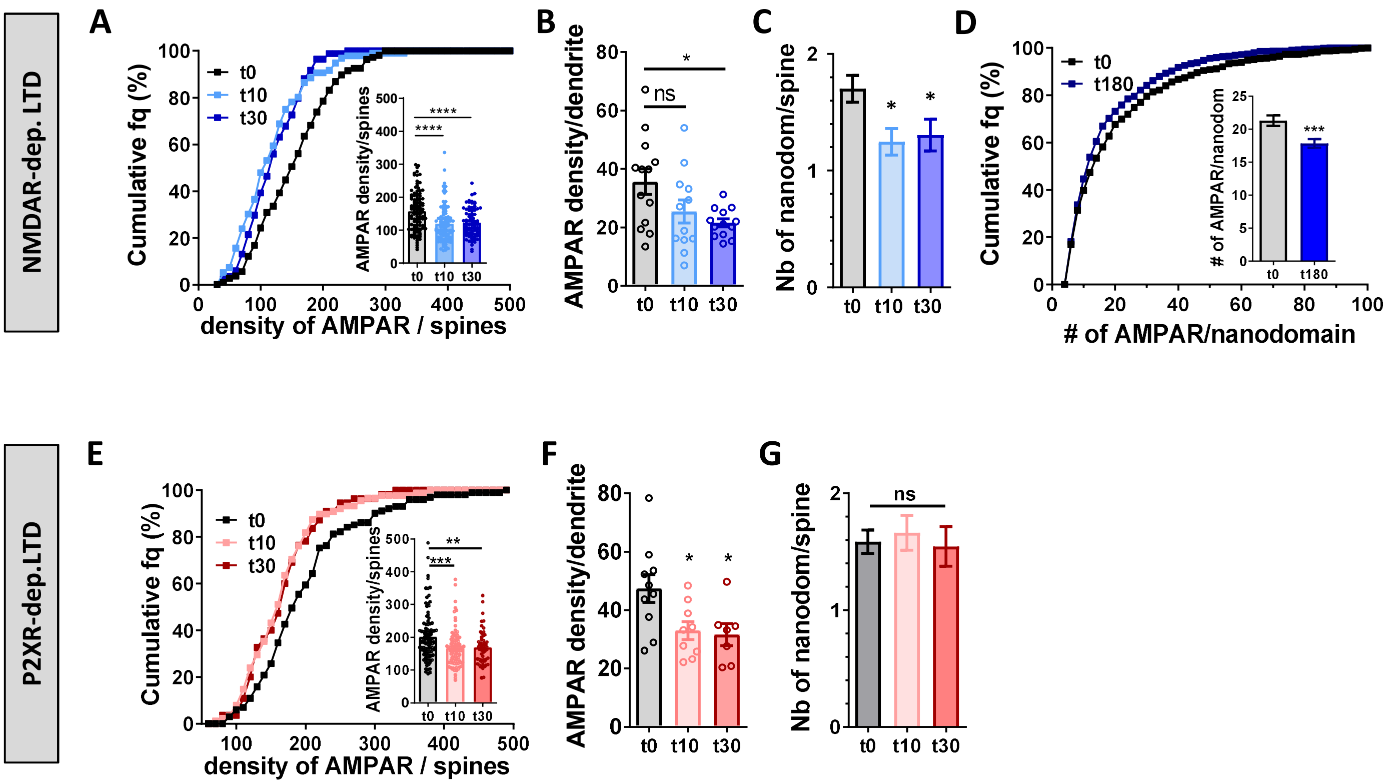

Supplementary Figure 1

Supplementary Figure 1. NMDA and ATP application triggers a rapid and long-lasting decrease of AMPAR surface expression and nanoscale organization.
(A) Cumulative distribution of AMPAR density per dendritic spine (n=107, 96 and 84 for t0, t10 and t30 respectively), and in the inset, the average histogram. The density of AMPARs per spine was measured 0, 10 and 30 minutes following NMDA treatment (mean +/- SEM, one-way ANOVA, $p<0.0001$ and Dunnett's post-test found significant differences between t0 and t10 or t30, $p<0.0001$). AMPAR surface expression is significantly decreased in dendritic spines 10 and 30 minutes following NMDA treatment compared to non-treated cells. (B) Average of AMPAR density per dendrite measured 0, 10 and 30 minutes following NMDA treatment (mean +/- SEM, n=13, 12 and 13 respectively, one-way ANOVA, $p=0.0183$ and Dunnett's post-test found significant difference between t0 and t30 $p=0.0118$ but not between t0 and t10, $p=0.0851$). AMPAR surface expression is significantly decreased in neuronal dendritic shafts 30 minutes following NMDA treatment compared to non-treated cells. (C) Average of AMPAR nanodomain number per dendritic spine measured 0, 10 and 30 minutes following NMDA treatment (mean +/- SEM, n=107, 105 and 79 respectively, one-way ANOVA, $p=0.0123$ and Dunnett's post-test found significant difference between t0 and t10 or t30, $p=0.0112$ and $p=0.0465$ respectively). AMPAR nanodomain number per dendritic spine is significantly decreased in neuronal dendritic shafts 10 and 30 minutes following NMDA treatment compared to non-treated cells. (D) Cumulative distribution of nanodomain AMPAR content (n=556 and 544 for t0 and t180 respectively), and in the inset, the average histogram. The number of AMPARs per nanodomains was measured at basal state (t0) and 180 minutes following NMDA treatment (mean +/- SEM, n=556 and 544, unpaired t-test, $p=0.0010$). Nanodomain content is significantly decreased 180 minutes following NMDA treatment compared to non-treated cells.
(E-G) Similar experiments as from A to C has been realized using ATP treatment to trigger LTD. (E) Cumulative distribution of AMPAR density per dendritic spine (n=101, 88 and 55 for t0, t10 and t30 respectively), and in the inset, the average histogram. The density of AMPARs per spines was measured 0, 10 and 30 minutes following ATP treatment (mean +/- SEM, one-way ANOVA, $p=0.0005$ and Dunnett's post-test found significant differences between t0 and t10 or t30, $p=0.0008$ and $p=0.0052$ respectively). AMPAR surface expression is significantly decreased in dendritic spines 10 and 30 minutes following ATP treatment compared to non-treated cells. (F) Average of AMPAR density per dendrite measured 0, 10 and 30 minutes following ATP treatment (mean +/- SEM, n=10, 9 and 7 respectively, one-way ANOVA, $p=0.0186$ and Dunnett's post-test found significant difference between t0 and t10 or t30 $p=0.0304$ and $p=0.0266$ respectively). AMPAR surface expression is significantly decreased in neuronal dendritic shafts 10 and 30 minutes following ATP treatment compared to non-treated cells. (G) Average of AMPAR nanodomain number per dendritic spine measured 0, 10 and 30 minutes following ATP treatment (mean +/- SEM, n=82, 68 and 55 respectively, one-way ANOVA, $p=0.8399$). AMPAR nanodomain number per dendritic spine is maintained 10 and 30 minutes following ATP treatment compared to non-treated cells.

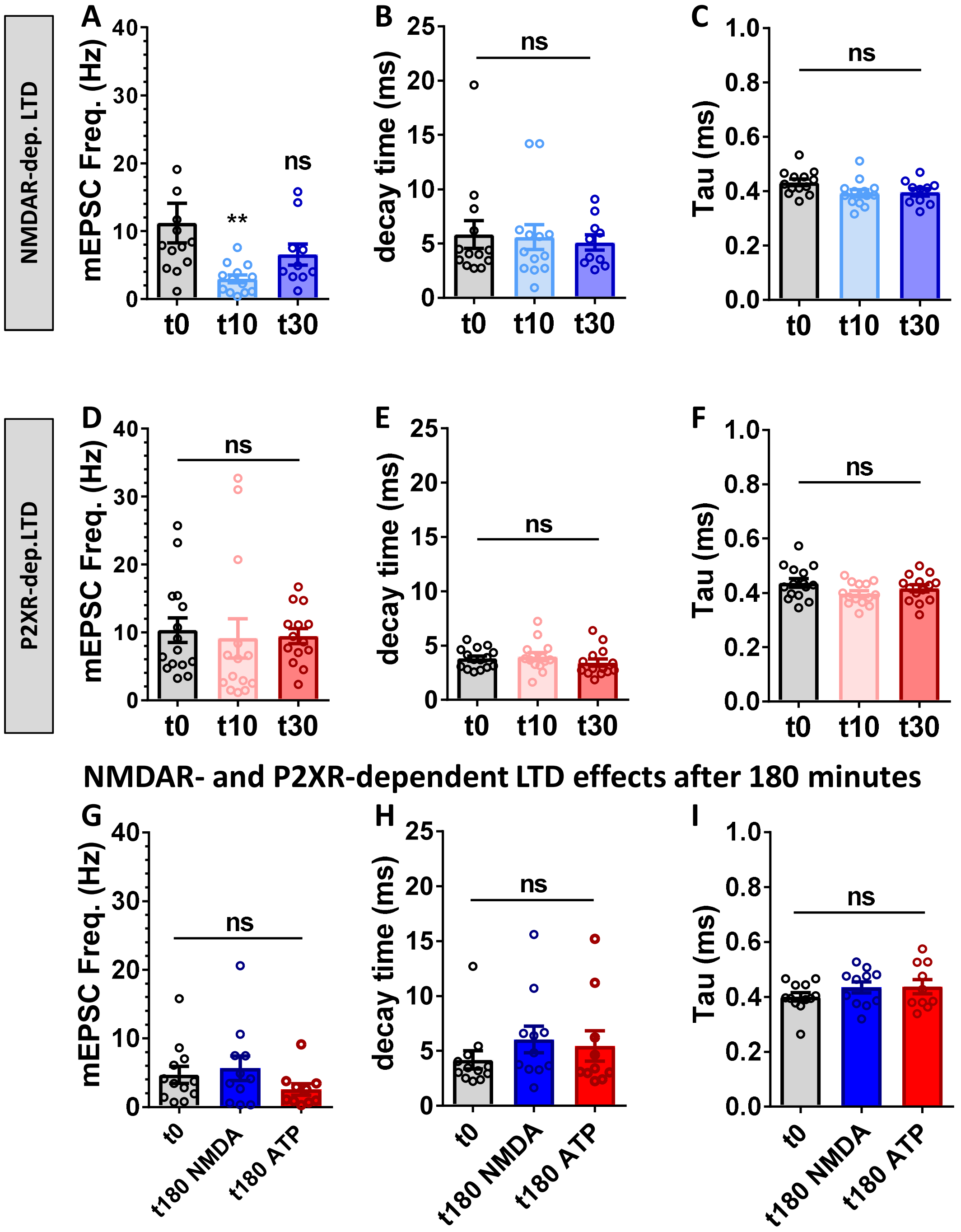

Supplementary Figure 2. Impact of NMDA and ATP treatment on mEPSC properties. (A-C) mEPSC frequency (A), decay time (B) and tau (C) at basal state (t0) or 10 and 30 minutes after NMDA treatment (mean +/- SEM, n=13, 13 and 10 respectively, (A) one-way ANOVA, p=0.0179 with Dunnett's post-test showing a significant difference between t0 and t10, p=0.0094; (B,C) one-way ANOVA, p=0.9033 and p=0.0707 respectively. (D-F) mEPSC frequency (D), decay time (E) and tau (F) at basal state (t0) or 10 and 30 minutes after ATP treatment (n=15, 14 and 14 respectively, one-way ANOVA, p=0.0905, p=0.4583 and p=0.1274 respectively). (G-I) mEPSC frequency (G), decay time (H) and tau (I) at basal state (t0) or 180 minutes after NMDA (t180 NMDA) or ATP (t180 ATP) treatments (n=12, 11 and 10 respectively, one-way ANOVA, p=0.2920, p=0.4848 and p=0.3639 respectively).

Supplementary Figure 2

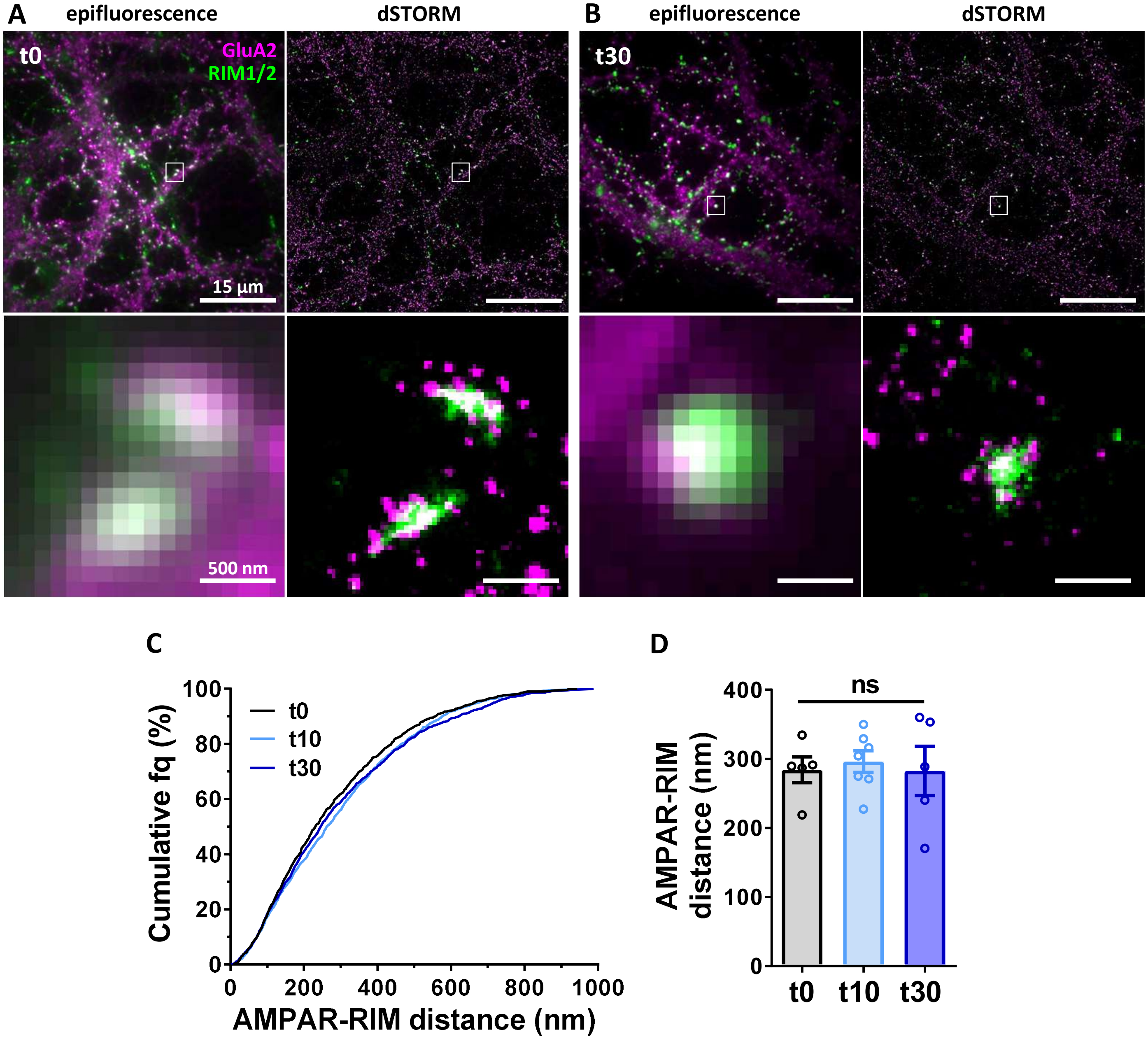

Supplementary Figure 3. NMDA treatment does not impact on the colocalization of the pre-synaptic RIM and the post-synaptic AMPAR domains

(A and B) Example of dual color D-STORM images with RIM1/2 in green and GluA2-containing AMPARs in purple before treatment (A) and 30 minutes after NMDA application (B). Top Right panels are low resolution images, top left panels are dSTORM reconstructed images, bottom are zoom on synapses from both low and high resolution images. Cumulative distribution (C) and Average per cell (D) of the AMPAR-RIM cluster distances (centroid to centroid) at t0 and 10 and 30 minutes following NMDA treatment. No significant differences are measured (one-way ANOVA, p=0.897). Scale bars for A and B top panels are 15µm and bottom panels are 500nm.

Supplementary Figure 3

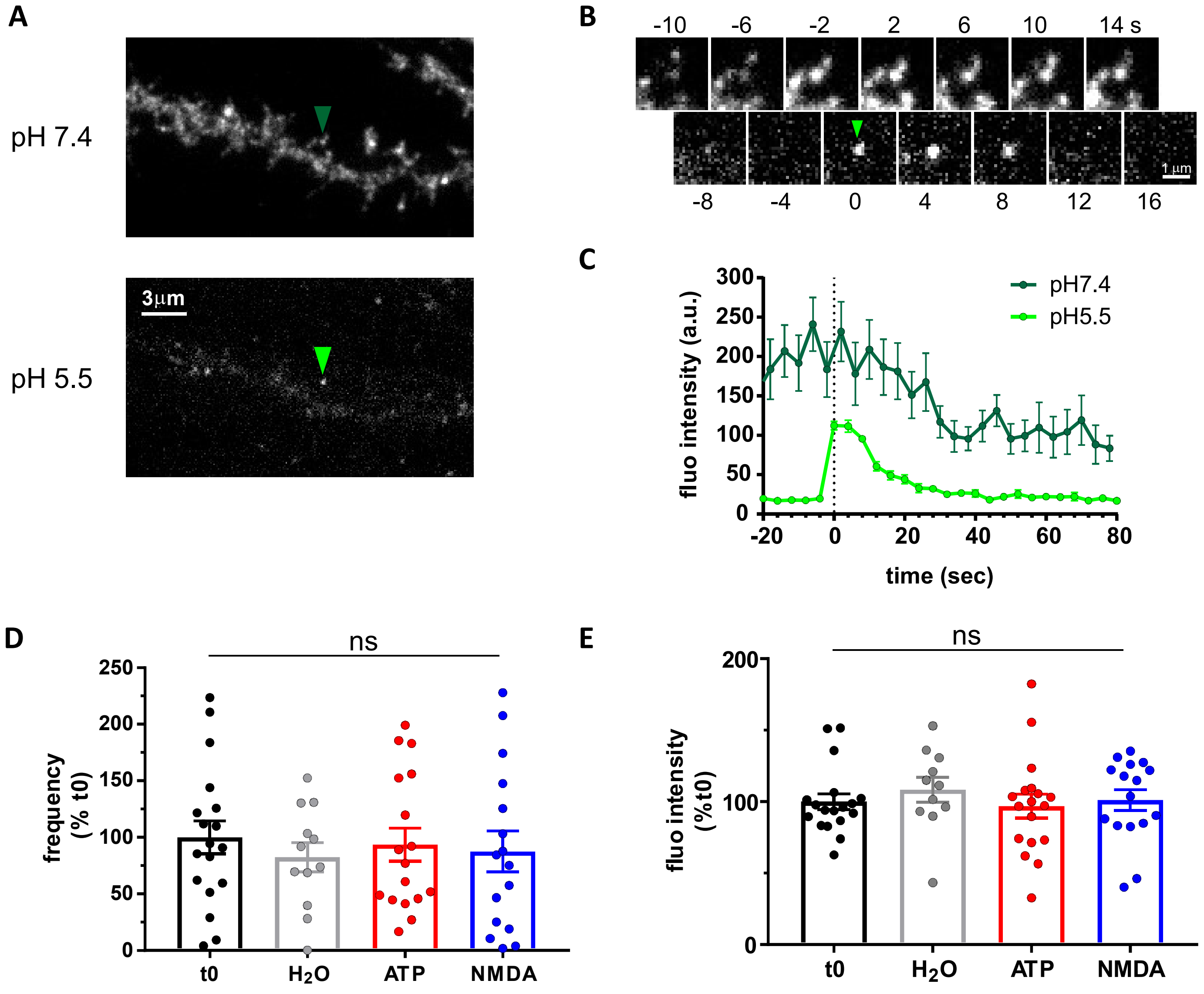

Supplementary Figure 4. NMDA or ATP do not alter endocytosis of AMPAR 3 hours post application.

(A) Dendrite of a neuron transfected with SEP-GluA1 and SEP-GluA2 and imaged with TIRF microscopy at pH 7.4 and pH 5.5 (4x contrast). Spots visible at pH 5.5 are intracellular receptors; green arrowhead highlights an endocytic vesicle with its corresponding cluster at pH 7.4. Scale bar = 3µm. (B) Example of the endocytic event, illustrated in A, detected with the ppH assay. Images taken at pH 7.4 (top) and 5.5 (bottom) at times relative to time 0, the moment of vesicle detection at pH 5.5. Scale bar = 1µm(C) Average fluorescence intensity (fluo.) of all the endocytic events detected in the cell shown in A, aligned to the time of vesicle detection (frame 0). (D) Frequency of endocytosis (in events.$min^{-1}.m^{-2}$) measured prior any stimulation (t0) or 3h after application of H_2O, ATP or NMDA, normalized to t0 of each session to correct for differences in basal activity of neuronal cultures; not normalized numbers are (mean +/- SEM): t0, 0.0157 ± 0.0034, n=18; H_2O, 0.0143 ± 0.0031, n=12; ATP, 0.0088 ± 0.0017, n=16; NMDA, 0.0146 ± 0.00286, n=17. (E) Average fluorescence intensity (fluo.) at time of vesicle formation of 621, 317, 264, 525 events in 18, 12, 16, 17 cells imaged prior any stimulation (t0), 3h after H_2O, ATP or NMDA application, respectively.

Supplementary Figure 4

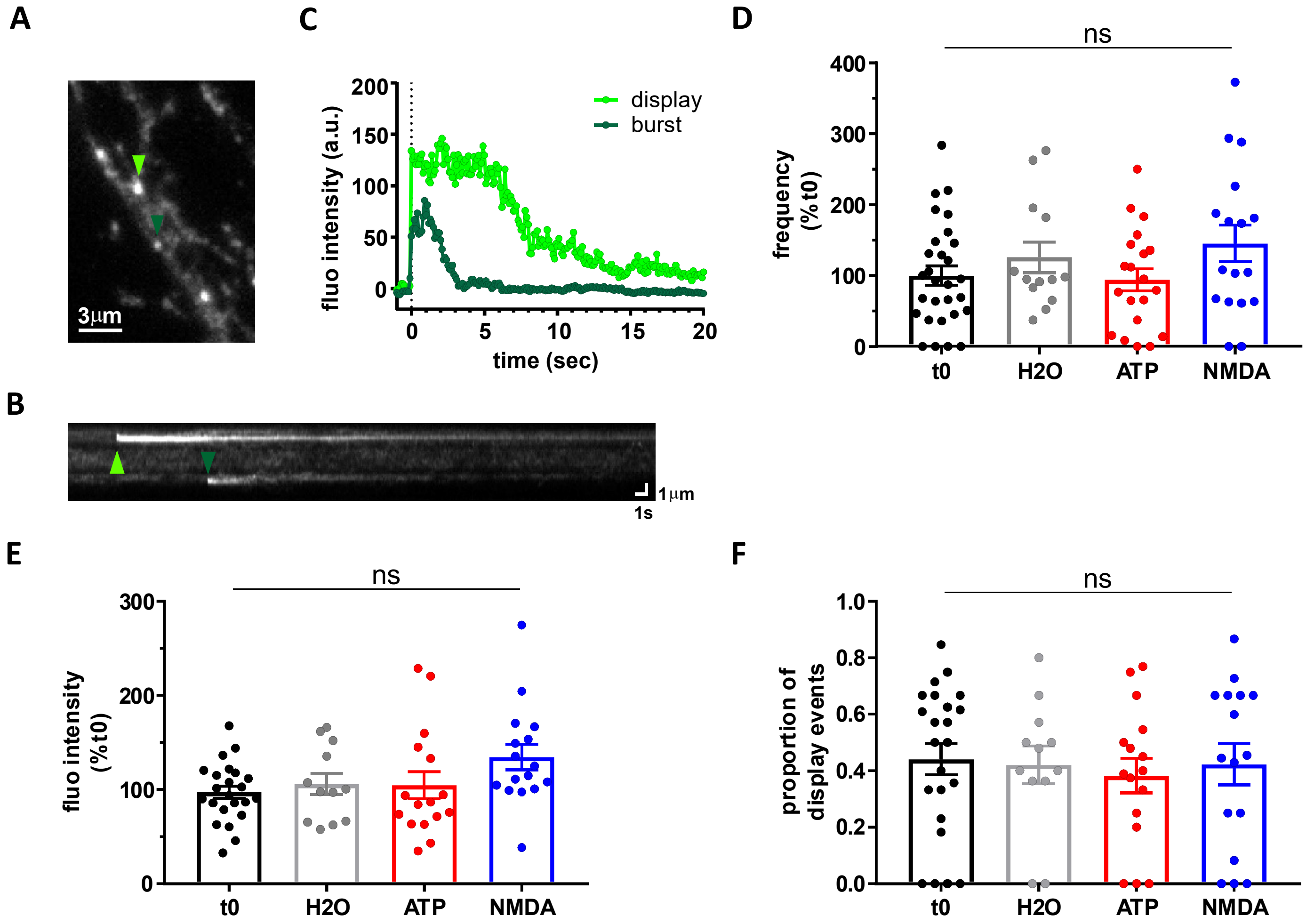

Supplementary Figure 5. NMDA or ATP do not alter exocytosis of AMPAR 3 hours post application.

(A) Dendrite of a neuron transfected with SEP-GluA1 and SEP-GluA2 and imaged with TIRF microscopy showing the two types of exocytic events: burst (dark green arrow) and display (light green arrow). (B) Kymograph from the line scan on the two events shown in B in which the display event remains visible for many seconds (light green arrow) and the burst event in which receptors quickly diffuse in the plasma membrane after exocytosis (light green arrow). (D) Fluorescence intensity of the two exocytic events from B, aligned to the time of detection (time 0). Note that fluorescence of burst event (dark green) decays to half of its maximal fluorescence within 2 seconds. (E) Frequency of exocytosis (in events.min^{-1}.m^{-2}) measured prior to any stimulation (t0) or 3h after application of H_2O, ATP or NMDA, normalized to t0 of each session to correct for differences in basal activity of neuronal cultures; not normalized numbers are: t0, 0.0242 ± 0.0037, n=29; H_2O: 0.0370 ± 0.0082, n=14; ATP, 0.0324 ± 0.0060, n=19; NMDA, 0.0257 ± 0.0043, n=17. (F) Average fluorescence intensity at time of detection of 183, 142, 148, 169 exocytic events in 23, 12, 16, 16 cells imaged prior any stimulation (t0), 3h after H_2O, ATP or NMDA application, respectively. (G) Proportion of display events from the same sample as in F.

Supplementary Figure 5

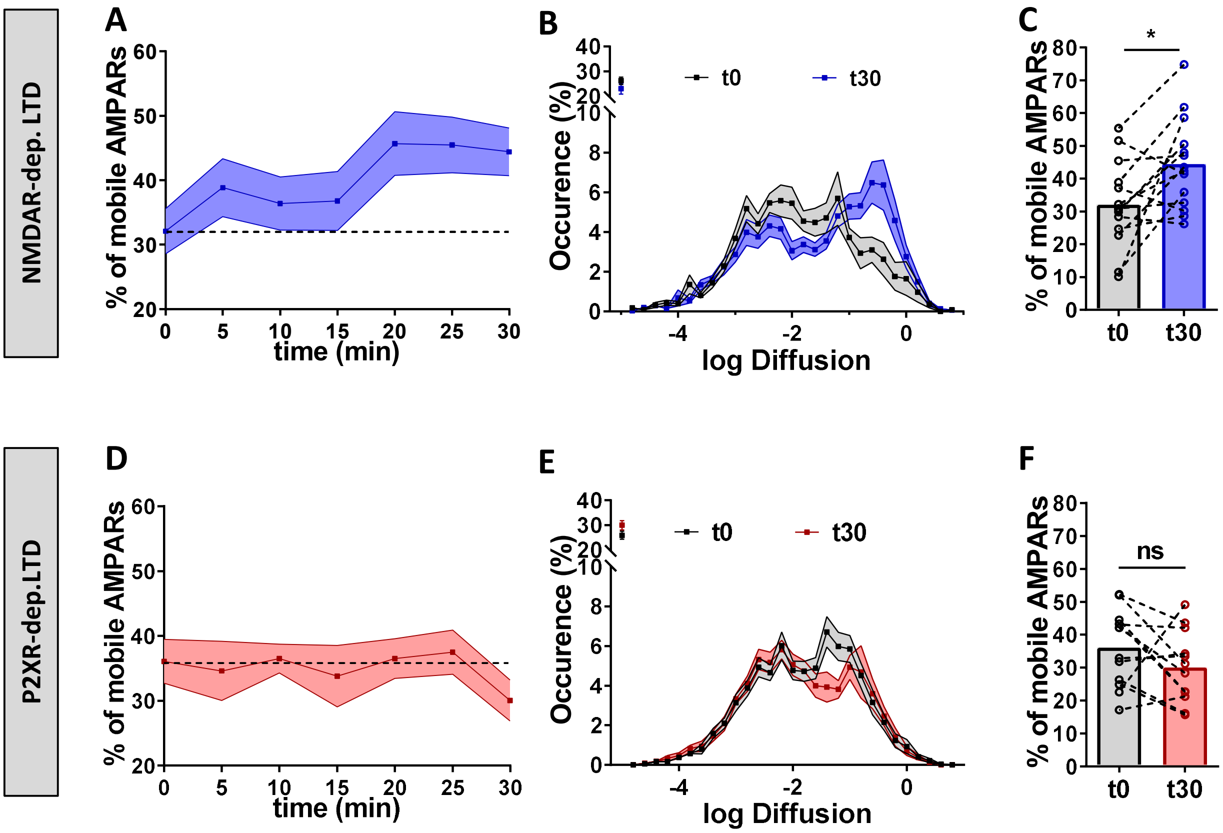

Supplementary Figure 6. NMDAR-dependent LTD but not P2XR-dependent LTD is associated to a long-term increase of synaptic AMPAR lateral diffusion.

(A) Time-lapse (from 0 to 30 minutes) of synaptic GluA2-containing AMPAR mobility following NMDAR-dependent LTD induction protocol (blue line) (mean +/- SEM, n=14). (B) Average distribution of the log(D), (D being the diffusion coefficient of endogenous AMPAR synaptic trajectories) in control condition (black line, n=14) and 30 minutes after NMDA treatment (blue line, n=14) (mean +/- SEM). (C) Average of the mobile fraction at synapses per cell, before and 30 minutes after NMDA treatment (n=14 cells, mean +/- SEM, paired t-test, p=0.0115). (D) Time-lapse (from 0 to 30 minutes) of synaptic GluA2-containing AMPAR mobility following P2XR-dependent LTD induction (red line) (mean +/- SEM, n=14). (E) Average distribution of the log(D) in control condition (black line, n=14) and 30 minutes after ATP treatment (red line, n=14) (mean +/- SEM). (F) Average of the mobile fraction at synapses per cell, before and 30 minutes after ATP treatment (n=14 cells, mean +/- SEM, paired t-test, p=0.1563).

Supplementary Figure 6

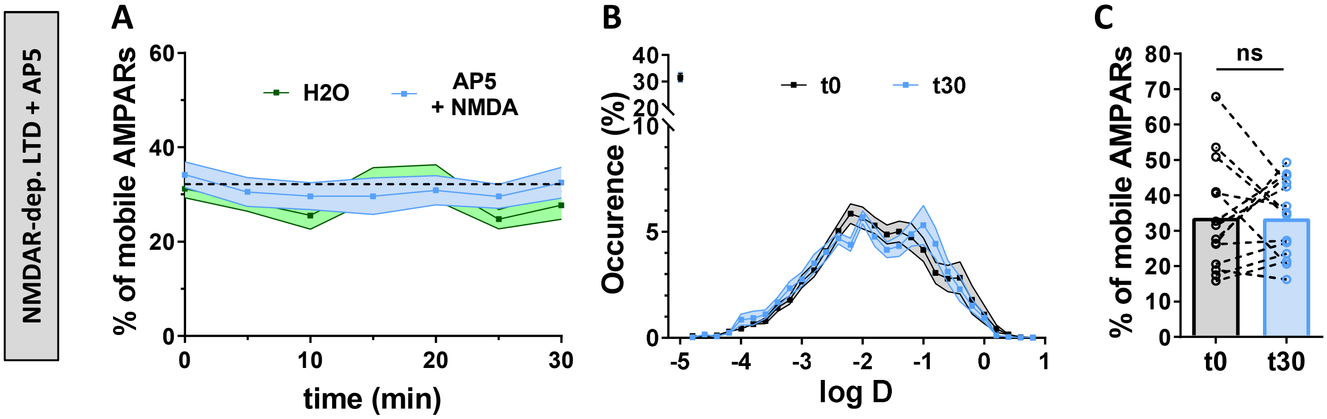

Supplementary Figure 7. AMPAR increased mobility after NMDA treatment is dependent of NMDAR specific activation.

(A) Time-lapse (from 0 to 30 minutes) of GluA2-containing AMPAR mobility following NMDAR-dependent LTD induction in the presence of AP5 (a specific NMDAR antagonist, light blue line) compared to vehicle application (green line) (n=12 and 10 respectively). No change in GluA2-containing AMPAR mobility occurs after NMDA application in the presence of AP5 (50 μM). (B) Average distribution of the log(D) in control condition (black line) and 30 minutes after NMDA treatment in presence of AP5 (light blue line). (C) Average of the mobile fraction per cell, before and 30 minutes after NMDA treatment with AP5 (n=14 cells, mean +/- SEM, paired t-test, p=0.9697).

Supplementary Figure 7

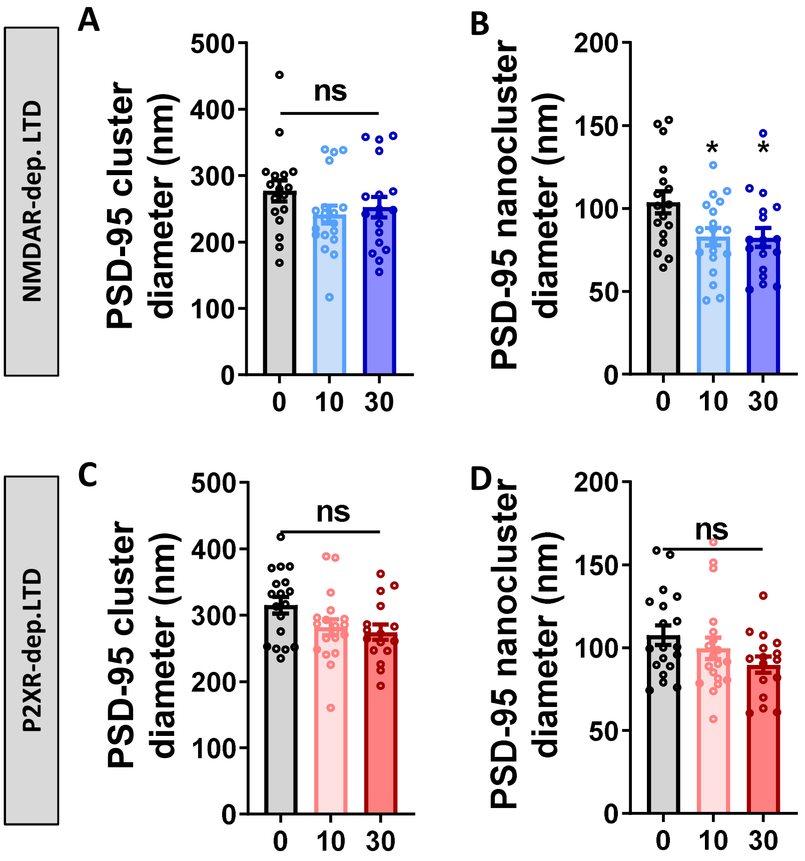

Supplementary Figure 8. Impact of NMDA or ATP treatment on PSD-95 cluster and nanocluster properties.

(A and B) Average size of PSD-95 molecules per cluster (A) and per nanoclusters (B) in basal state, 10 and 30 minutes after NMDA treatment (mean +/- SEM, n=17, 19 and 18 respectively, one-way ANOVA p=0.2338 for clusters; one-way ANOVA p=0.0212 and Dunnett's post-test found significant between t0 and t10 and between t0 and t30 conditions, p=0.0297 and p=0.0268 respectively, for nanoclusters). (C and D) Average size of PSD-95 molecules per cluster (C) and per nanoclusters (D) in basal state, 10 and 30 minutes after ATP treatment (mean +/- SEM, n=18, 19 and 16 respectively, one-way ANOVA p=0.0504 for clusters; one-way ANOVA p=0.1165 for nanoclusters).

Supplementary Figure 8

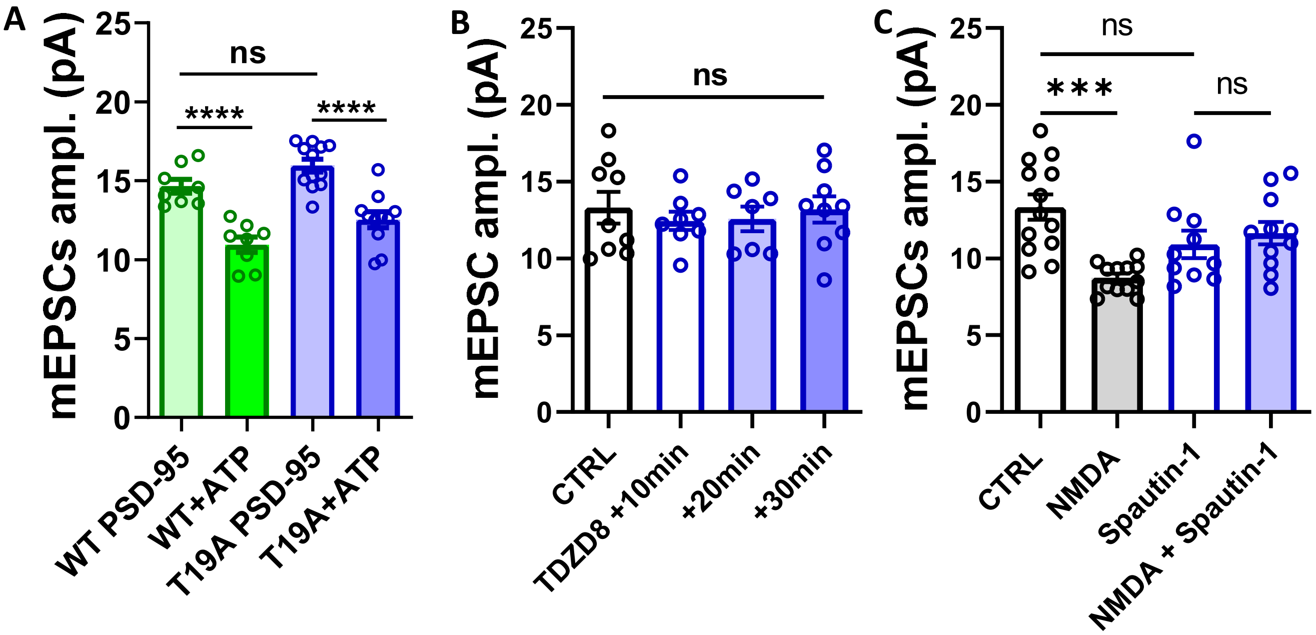

Supplementary Figure 9. PSD95 degradation by autophagy is specific of NMDAR-dependent LTD.

(A) Average of the mESPC amplitude recorded on neurons expressing WT or T19A mutant PSD-95, 0 and 30 minutes after ATP treatment. Both neuron types present a significant decrease of mEPSC amplitude following LTD induction (mean +/- SEM, one-way ANOVA, $p<0.0001$ and Tukey's post-test results are realized between each conditions, N=8, 8, 12, 11). (B) Average of the mESPC amplitude recorded on neurons, 0, 10, 20 and 30 minutes after TDZD8 application. The presence of TDZD8 does not affect mEPSC amplitude (mean +/- SEM, one-way ANOVA, $p<0.0001$ and Tukey's post-test results are realized between each conditions, N=9, 8, 7, 9). (C) Average of the mESPC amplitude recorded on neurons, before and after induction of LTD by NMDA application, in the absence or the presence of spautin (an autophagy inhibitor, 10 µM). The presence of spautin suppresses the LTD induction (mean +/- SEM, one-way ANOVA, $p<0.001$ and Tukey's post-test results are realized between each conditions, N=13, 12, 10, 11).

Supplementary Figure 9

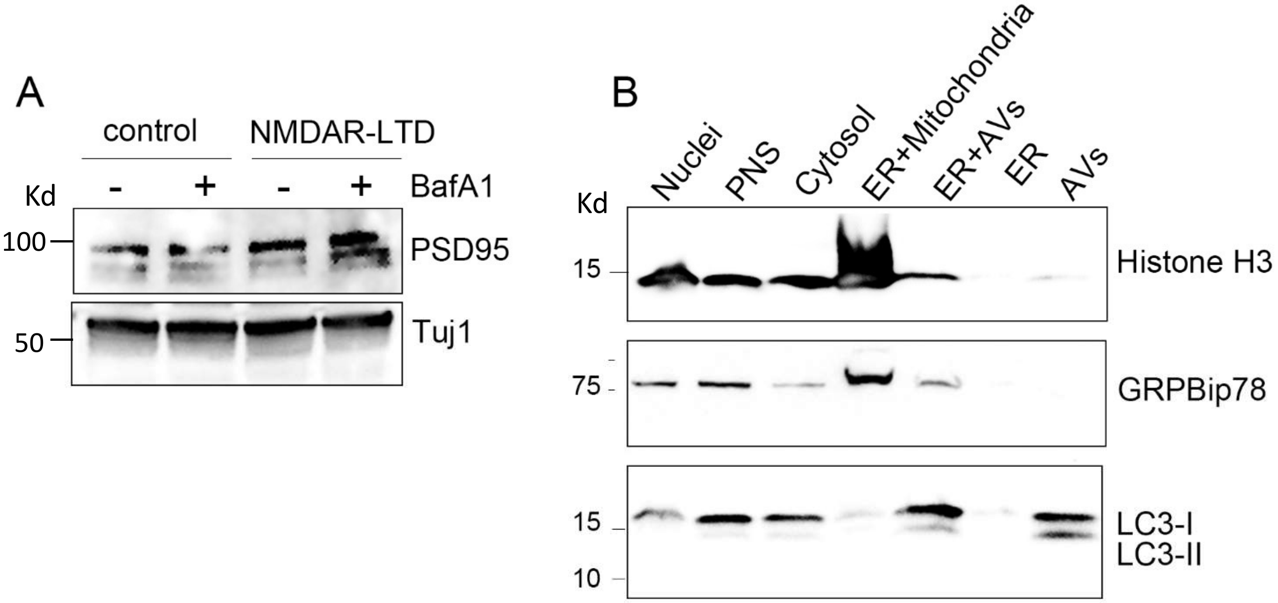

Supplementary Figure 10. Validation of the autophagic vesicle purification quality.
(A) Western blot analysis for PSD95 and the loading control b-III tubulin (Tuj1) in lysates of cultured control neurons or one hour after NMDAR-dependent LTD, in the presence or absence of Bafilomycin A1 (BafA1). BafA1 was applied fifteen minutes before, during the LTD induction and for one hour after it. The same total duration was used for the application of BafA1 in the control neurons. Note the accumulation of PSD95 in the BafA1-treated neurons after LTD induction.(B) Western blot analysis of the fractions collected during the autophagic vesicle purification procedure for an ER marker (GRP97Bip), a nuclear marker (Histone-H3) and an autophagic vesicle marker (LC3B-II). Note the absence of ER and nuclear markers from the final AV purified sample, which is instead enriched for LC3B-II.

Supplementary Figure 10

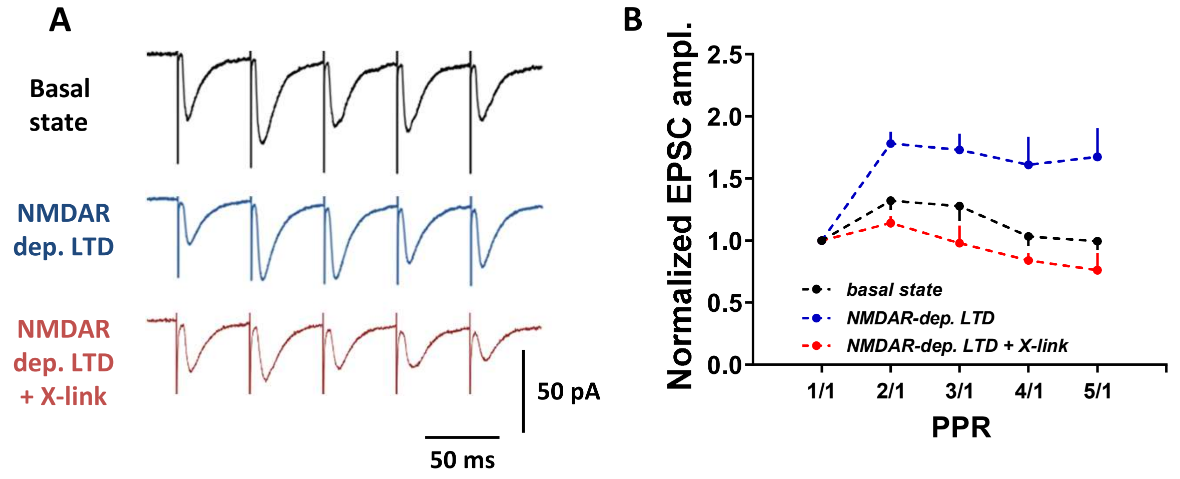

Supplementary Figure 11.

(A) Average of the 5 EPSC amplitudes, normalized by the first response intensity. Paired-pulse stimulation was performed on acute hippocampal slices either untreated (basal state, n=9), or 30 minutes after NMDAR-dependent LTD induction in presence of anti-GFP antibody (control, n=9) or anti-GluA2 antibody (0.3µg/µL, inducing AMPAR cross-link, n=2). Injection of antibodies have been done in the stratum radiatum area of the whole-cell patch neuron 20 minutes after the NMDAR-dependent LTD induction (mean +/- SEM). As described in Heine et al. 2008 and Constals et al. 2015, GluA2 cross-linking decrease the PPR.

Supplementary Figure 11

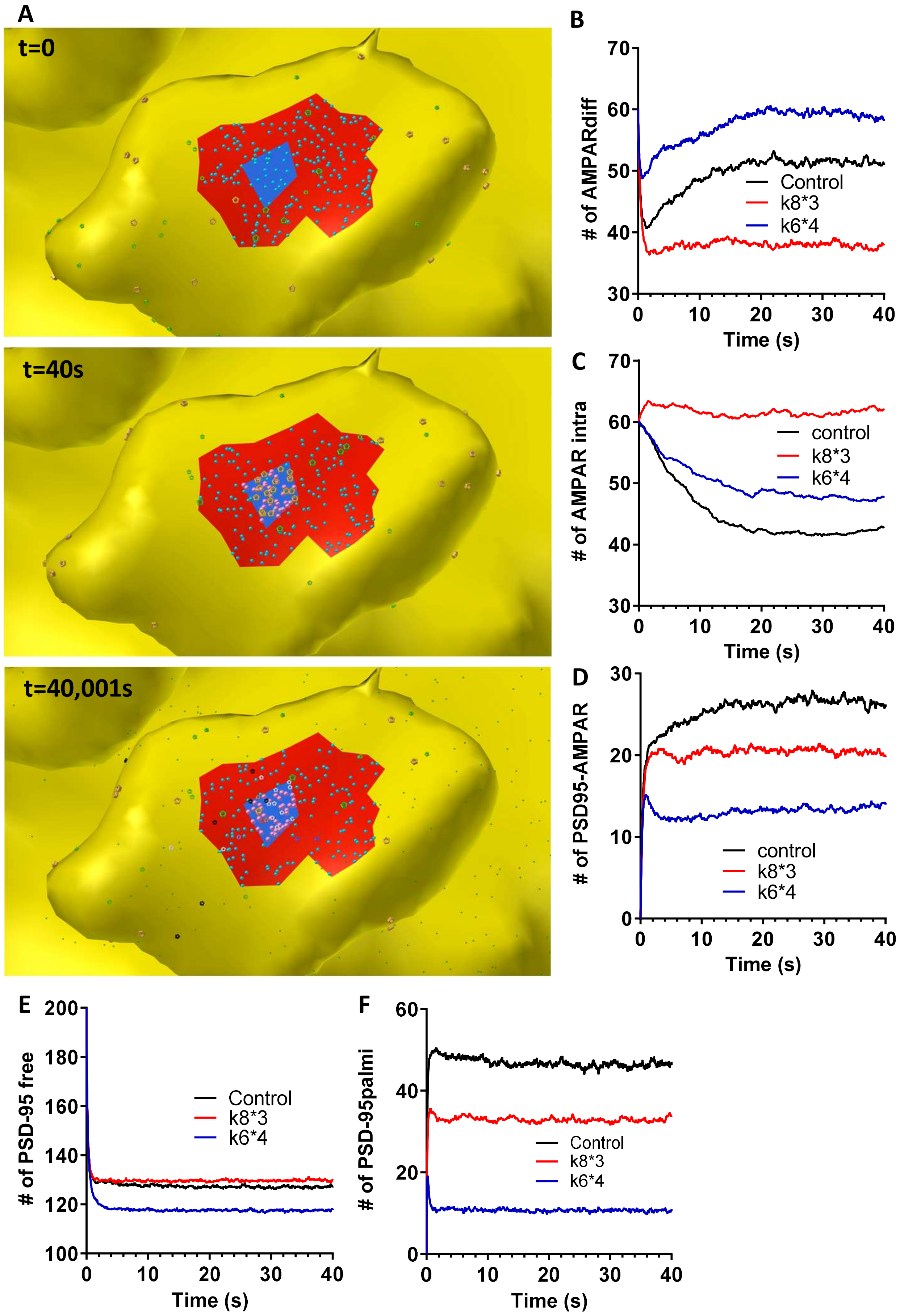

Supplementary Figure 12

Supplementary Figure 12. Effect of *in silico* LTD on the equilibrium between various state of both PSD-95 and AMPAR

(A) Example of images obtained with the model at t=0 (top panel), t=40 s when protein organization reach a stable state (middle panel) and at t=40.001 s, 1 ms after first glutamate release (bottom panel). Icosahedrons represent AMPAR, and colors differ in function of their states: orange for the closed, green for the endocytosed, white for the opened, black for the desensitized, etc. Dots represent PSD-95, blue for the freely diffusive and pink for the palmitoylated. (B-F) Kinetics of accumulation of the various protein species in control or when LTD is mimicked by either an increase of endocytosis (red line, k8*3) or by an inactivation of PSD-95 (blue line, k6*4). We report, the evolution of the number of diffusive AMPAR (B), internalized AMPAR (C), PSD-95 coupled to AMPAR (D), free PSD-95 (E) and palmitoylated PSD-95 (F). The proportion of each species at the equilibrium are closed to the values experimentally obtained.

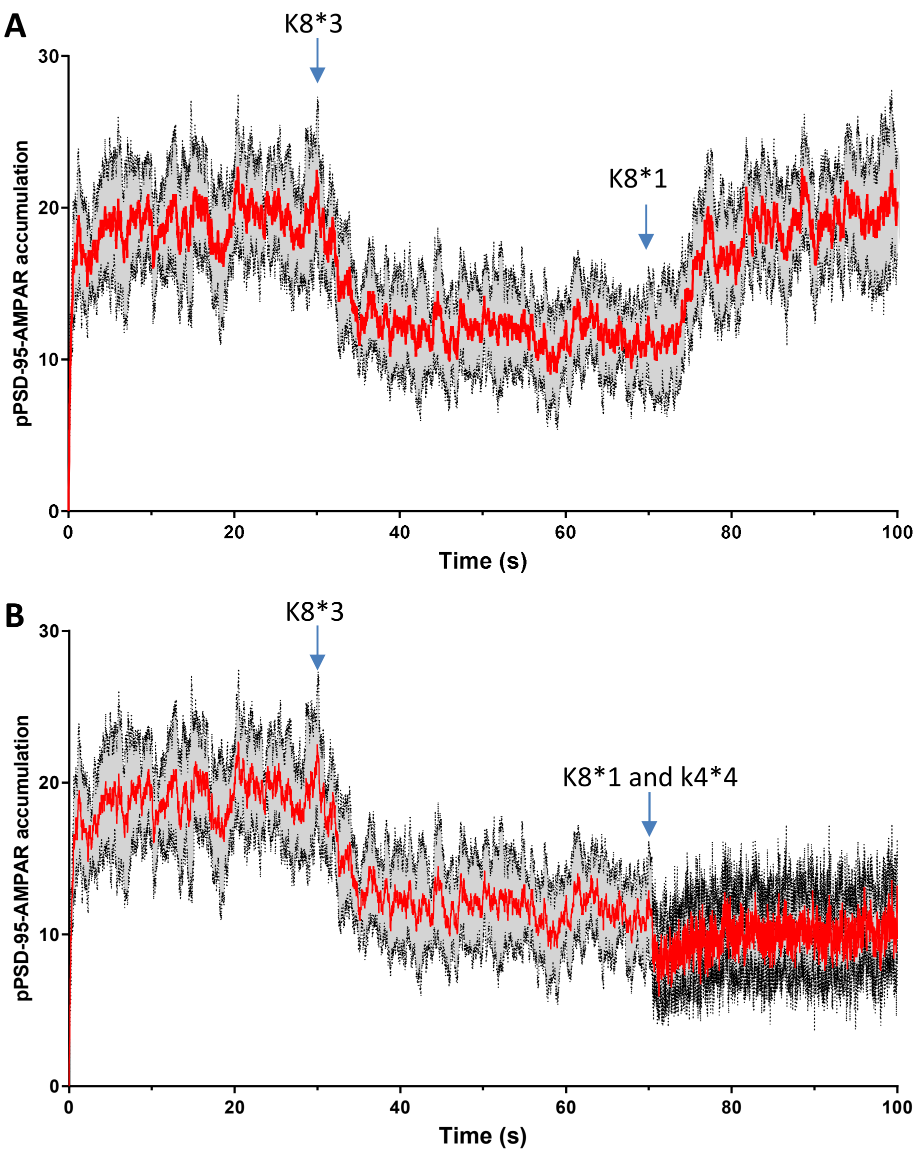

Supplementary Figure 13. Simulation shows that LTD-induced increase of endocytosis needs to be maintained or compensate to stabilize depression of AMPAR

(A) Simulation of AMPAR accumulation in nanodomain. For the first 30 s we observe the recruitment of AMPAR. At 30 s, the endocytosis rate is multiplied by 3 to mimic an LTD. At 70 s the endocytosis rate is returned to its initial value, triggering to a progressive replenishment of the AMPAR nanodomain. (B) Similar simulations are realized but at 70 s, we re-initiate endocytosis rate and in parallel we decreased the affinity of AMPAR for the traps (as shown Figure 6C). We observed a stabilization of the nanodomain depletion, and interestingly, to an increase of the noise due to the more rapid exchange of AMPAR.

Supplementary Figure 13
