# Supplementary material for: NMDAR-dependent long-term depression is associated with increased short term plasticity through autophagy mediated loss of PSD-95
Source: Nat Commun. 2021 May 14;12:2849. doi: 10.1038/s41467-021-23133-9 (PMC8121912; doi:10.1038/s41467-021-23133-9)
Supplement: Supplementary file 3 — Reporting Summary [file 41467_2021_23133_MOESM3_ESM.pdf]

nature research

Corresponding author(s): eric hosy
Last updated by author(s): Mar 10, 2021

Reporting Summary

Nature Research wishes to improve the reproducibility of the work that we publish. This form provides structure for consistency and transparency in reporting. For further information on Nature Research policies, see our Editorial Policies and the Editorial Policy Checklist.

Statistics

For all statistical analyses, confirm that the following items are present in the figure legend, table legend, main text, or Methods section.

n/a	Confirmed	
☐	☒	The exact sample size (*n*) for each experimental group/condition, given as a discrete number and unit of measurement
☐	☒	A statement on whether measurements were taken from distinct samples or whether the same sample was measured repeatedly
☐	☒	The statistical test(s) used AND whether they are one- or two-sided<br>*Only common tests should be described solely by name; describe more complex techniques in the Methods section.*
☐	☒	A description of all covariates tested
☐	☒	A description of any assumptions or corrections, such as tests of normality and adjustment for multiple comparisons
☐	☒	A full description of the statistical parameters including central tendency (e.g. means) or other basic estimates (e.g. regression coefficient) AND variation (e.g. standard deviation) or associated estimates of uncertainty (e.g. confidence intervals)
☐	☒	For null hypothesis testing, the test statistic (e.g. F, t, r) with confidence intervals, effect sizes, degrees of freedom and P value noted<br>*Give P values as exact values whenever suitable.*
☒	☐	For Bayesian analysis, information on the choice of priors and Markov chain Monte Carlo settings
☒	☐	For hierarchical and complex designs, identification of the appropriate level for tests and full reporting of outcomes
☒	☐	Estimates of effect sizes (e.g. Cohen's *d*, Pearson's *r*), indicating how they were calculated

Our web collection on statistics for biologists contains articles on many of the points above.

Software and code

Policy information about availability of computer code

Data collection	For all imaging acquisition, Metamorph 7.8 64X(PalmTracer plugin) was used.<br>For all electrophysiological recordings, HEKA patch master EPC 10 was used.<br>Computer modeling was performed using MCell/CellBlender simulation environment (http://mcell.org) with MCell version 3.3, CellBlender version 1.1, and Blender version 2.77a (http://blender.org)
Data analysis	Excel, Graphpad Prism6 and Metamorph 7.8 64X(PalmTracer plugin) softwares were mostly used.<br>The matlab script used for mEPSC analysis is available on MATLAB File Exchange, ID: 61567; http://uk.mathworks.com/matlabcentral/fileexchange/61567-peaker-analysis-toolbox.<br>StimFit (https://doi.org/10.3389/fninf.2014.00016) was also used for electrophysiology analysis.

For manuscripts utilizing custom algorithms or software that are central to the research but not yet described in published literature, software must be made available to editors and reviewers. We strongly encourage code deposition in a community repository (e.g. GitHub). See the Nature Research guidelines for submitting code & software for further information.

Data

Policy information about availability of data

All manuscripts must include a data availability statement. This statement should provide the following information, where applicable:

- Accession codes, unique identifiers, or web links for publicly available datasets
- A list of figures that have associated raw data
- A description of any restrictions on data availability

The datasets generated during and/or analysed during the current study are available from the corresponding author on reasonable request.

Field-specific reporting

Please select the one below that is the best fit for your research. If you are not sure, read the appropriate sections before making your selection.

[x] Life sciences [ ] Behavioural & social sciences [ ] Ecological, evolutionary & environmental sciences

For a reference copy of the document with all sections, see nature.com/documents/nr-reporting-summary-flat.pdf

Life sciences study design

All studies must disclose on these points even when the disclosure is negative.

Sample size	Experiments are classical electrophysiology or imaging experiments. The samples size is empirically determined with always at least three independent dissections.
Data exclusions	Outliers were detected by Grubbs' test and removed from the graph and the analysis.
Replication	All experiments were obtained from at least 3 independent experiments.<br>Attempts of replication have been done by another experimenter for results obtained in Figure 1, 2 and 3. All attempts at replication were successful.
Randomization	All experiments were randomly allocated into experimental groups.
Blinding	Experimenters were blinded to the treatment (NMDA, ATP or vehicle) to apply to samples; as well as during analysis.

Reporting for specific materials, systems and methods

We require information from authors about some types of materials, experimental systems and methods used in many studies. Here, indicate whether each material, system or method listed is relevant to your study. If you are not sure if a list item applies to your research, read the appropriate section before selecting a response.

Materials & experimental systems

n/a	Involved in the study	
[ ]	[x]	Antibodies
[x]	[ ]	Eukaryotic cell lines
[x]	[ ]	Palaeontology and archaeology
[ ]	[x]	Animals and other organisms
[x]	[ ]	Human research participants
[x]	[ ]	Clinical data
[x]	[ ]	Dual use research of concern

Methods

n/a	Involved in the study	
[x]	[ ]	ChIP-seq
[x]	[ ]	Flow cytometry
[x]	[ ]	MRI-based neuroimaging

Antibodies

Antibodies used	monoclonal mouse anti-GluA2 antibody (mouse antibody, provided by E. Gouaux, Portland, USA)<br>monoclonal mouse anti-PSD-95 antibody (MA1-046, ThermoFischer, clone 7E3-1B8)<br>polyclonal rabbit anti-RIM 1/2 antibody (synaptic systems, 140 203)<br>Alexa 532 coupled anti-mouse IgG secondary antibodies (ThermoFisher, A21235)<br>Alexa 647 coupled anti-rabbit IgG secondary antibodies (ThermoFisher, A21244)<br>Alexa 647 coupled anti-mouse IgG secondary antibodies (ThermoFisher, A21235)<br>Alexa 488 coupled anti-mouse IgG secondary antibodies (ThermoFisher, A11001).<br>polyclonal rabbit anti-T19PSD-95 (Abcam, ab16496)<br>monoclonal mouse anti-p62 (Abcam, ab56416)<br>Polyclonal rabbit LC3 primary antibody (sigma aldrich, L8918)

	GRP78Bip (ab21685, Abcam)
Validation	Monoclonal mouse anti-GluA2 antibody (mouse antibody, provided by E. Gouaux, Portland, USA) has been validated on neuronal culture of GluA2-KO mice, see also Giannone et al 2010 (DOI: 10.1016/j.bpj.2010.06.005), Nair et al 2013 (DOI: 10.1523/JNEUROSCI.2381-12.2013)<br>For all other antibodies information are available on the manufacturer website.

Animals and other organisms

Policy information about studies involving animals; ARRIVE guidelines recommended for reporting animal research

Laboratory animals	Dissociated Hippocampal neurons were cultured from Sprague Dawley rat embryos (E18).<br>Acute slices were prepared from P16-18 Sprague-Dawley rats of both sexes.<br>Autophagic vesicle preparation were obtained from hippocampal sections of 5 C57BL/6 adult mice of both sexes.<br>darkl-ight cycle: 12/12; humidity: 40%; temperature: 20°C
Wild animals	no wild animals has been used in this study
Field-collected samples	no field-collected samples have been use in this study
Ethics oversight	All experiments were performed in accordance with the guidelines established by the European Communities Council (Directive 2010/63/EU of September 22, 2010) and were approved by the Animal Experimental Committee of Bordeaux.

Note that full information on the approval of the study protocol must also be provided in the manuscript.
